# An investigation of female genital schistosomiasis and associated genital infections in Southern Malawi

**DOI:** 10.1017/S0031182025100802

**Published:** 2025-12

**Authors:** Dingase Kumwenda, Sekeleghe Kayuni, Guilleary Deles, Bright Mainga, Lilly Atkins, Fatima Ahmed, Abbigail Cawley, Lucas J. Cunningham, David Lally Jnr, Priscilla Chammudzi, Donales Kapira, Gladys Namacha, Alice Chisale, Tereza Nchembe, Louis Kinley, Ephraim Chibwana, Gilbert Chapweteka, Henry Chibowa, Victor Kumfunda, Alexandra Juhasz, Sam Jones, Ruth Cowlishaw, John Archer, Angus M. O'Ferrall, Sarah Rollason, Andrew Nguluwe, John Chiphwanya, Holystone Kafanikhale, Peter Makaula, E. James LaCourse, J. Russell Stothard, Janelisa Musaya

**Affiliations:** 1 Malawi Liverpool Wellcome Research Programme, Kamuzu University of Health Sciences, Blantyre, Malawi; 2 Department of Tropical Disease Biology, Liverpool School of Tropical Medicine, Merseyside, UK; 3 Obstetrics and Gynaecology Department, Queen Elizabeth Central Hospital, Blantyre, Malawi; 4 Laboratory Department, Mangochi District Hospital, Mangochi, Malawi; 5 Radiology Department, Queen Elizabeth Central Hospital, Blantyre, Malawi; 6 Nsanje District Hospital, Ministry of Health, Nsanje, Malawi; 7 Mangochi District Hospital, Ministry of Health, Mangochi, Malawi; 8 Institute of Medical Microbiology, Semmelweis University, Budapest, Hungary; 9 School of Biosciences, Cardiff University, Cardiff, UK; 10 National Schistosomiasis and Soil-Transmitted Helminths Control Programme, Community Health Sciences Unit, Ministry of Health, Lilongwe, Malawi; 11 Pathology Department, School of Medicine and Oral Health, Kamuzu University of Health Sciences, Blantyre, Malawi

**Keywords:** cervicovaginal lavage, FGS, hybrid, Lake Malawi, *Schistosoma haematobium*, *Schistosoma mattheei*, Shire River

## Abstract

Urogenital schistosomiasis (UGS) caused by zoonotic or hybrid schistosome infection(s) is an emerging public health concern in Malawi, and we describe a 1-year clinical sub-study with 3 inspection time points for female genital schistosomiasis (FGS) upon selecting 86 women with proven UGS. This sub-study was set within a broader 2-year longitudinal ‘Hybridization in UroGenital Schistosomiasis (HUGS)’ investigation. A detailed cervicovaginal examination with a portable colposcope was conducted, examining cervicovaginal lavage (CVL), cervical swab, cervical biopsy and urine with traditional parasitological and molecular diagnostic methods. At baseline, overt FGS by colposcopy was 72.1%, 64.3% by CVL real-time PCR and 51.2% by both colposcopy and CVL-PCR, noting urine-microscopy could often be negative. Human papillomavirus was detected in 31.0% of the cervical swabs, with 8.3% women also FGS positive by colposcopy and real-time PCR. Over the year, FGS prevalence by colposcopy increased by 32.7% during the study to 84.6%, homogenous yellow and grainy sandy patches being very common in the youngest 18–25 age group, where 51.9% were positive. FGS appears widespread locally and we discuss difficulties in its detection without invasive sampling. In addition to the presence of *S. haematobium*, S. *mattheei* was noted alongside key concurrent sexually transmitted infections. From our findings, we point out that improved prevention and management of FGS is required, foremost, better availability and regular accessibility to praziquantel treatment is needed. Furthermore, targeted health education, raised community awareness and dovetailing synergistic public health activities within Sexual and Reproductive Health services and local HIV/AIDS programmes could develop an appropriate holistic health intervention package.

## Graphical abstract

**Figure:**
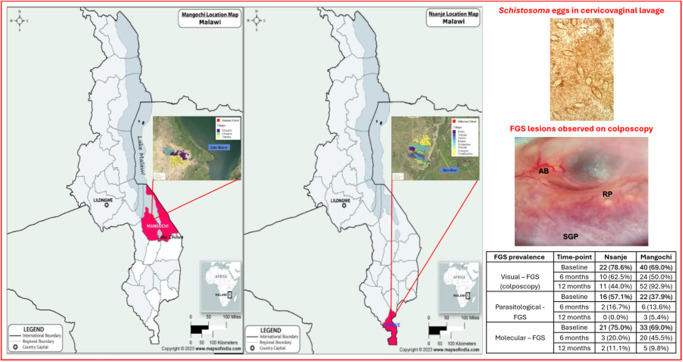

## Introduction

Across much of sub-Saharan Africa (SSA), female genital schistosomiasis (FGS), a neglected gynaecological tropical disease, results from chronic infection with schistosome worms predominantly *Schistosoma haematobium* which causes urogenital schistosomiasis (UGS) (Bustinduy et al. 2022; Orish et al. 2022; Buonfrate et al. 2025). It is a significant public health concern, affecting some 56 million women, and leads to enduring genital morbidity often with detrimental life-changing events (Patel et al. 2021; Kayuni et al. 2024a). In Malawi, zoonotic (*Schistosoma mattheei*) or hybrid (*S. haematobium × mattheei*) schistosome infection(s) is an emerging public health concern (O’Ferrall et al. 2025), yet their association and infection dynamics with FGS are not yet clear.

Clinical FGS manifests as granulomas, sandy patches and abnormal blood vessels (ABV) in the female genital tract (WHO, 2015), often resulting in symptoms such as vaginal discharge, abnormal genital bleeding, pelvic pain and infertility, mimicking symptoms of sexually transmitted infections (STIs) (Govender et al. 2024). Despite its prevalence and owing to short comings in primary health surveillance and care, FGS remains underdiagnosed and undertreated across much of SSA (WHO, 2015; Bustinduy et al. 2022; Ndubani et al. 2024).

In Southern Malawi, a recent clinical research study of women of reproductive age has shown clinical FGS to range from 8.0% to 27.0% using different diagnostic techniques and has demonstrated substantial morbidity in certain individuals (Lamberti et al. 2024a). The origins of FGS, like UGS, however, likely commence in a younger age, for example in adolescence as girls are frequently exposed to infested water during their domestic chores, agricultural activities and recreational events, facilitating the transmission of *S. haematobium* (Makaula et al. 2014; Nyangulu et al. 2022).

In addition to FGS, sexually active adolescent girls and women are at risk of various genital infections (Dehne and Riedner, 2001; WHO, 2005), which may coexist with or be exacerbated by UGS (Kjetland et al. 2008; Shukla et al. 2023; Søfteland et al. 2024). The interplay between FGS and these coinfections poses a significant challenge to women’s wellbeing and reproductive health, leading to an increased susceptibility to HIV, poor pregnancy outcomes and long-term complications (Rafferty et al. 2021; Govender et al. 2024). Understanding the distribution and co-occurrence of FGS with other genital infections, is essential to improving diagnostic accuracy and guiding effective intervention strategies. Despite its clinical and public health importance, FGS remains understudied in Malawi, and the associations between FGS and other genital infections are not well understood.

As part of a larger community-based study in 2 selected districts of Southern Malawi, funded by National Institute for Health Research (NIHR) and Wellcome Trust UK, entitled ‘Hybridization in UroGenital Schistosomiasis’ (HUGS), a year-long FGS sub-study conducted between 2023 and 2024. This study aimed to investigate the prevalence of FGS associated with human, zoonotic and hybrid schistosome species and its association with other presenting genital infections among women in Southern Malawi.

## Materials and methods

### Study area, design and participants

**Figure 1.**
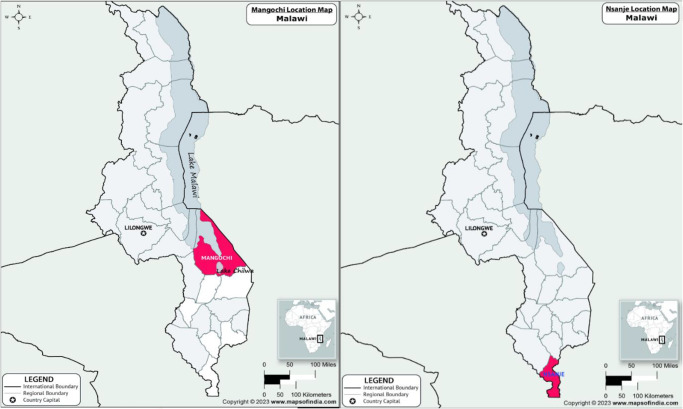
Map showing 2 study communities around Samama school in Mangochi District and Mthawira school in Nsanje District of Southern Malawi where participants originated.

This 1-year longitudinal cohort study was conducted in 2 study sites of Mthawira community from Nsanje District along the margins of Shire River (S 16.849802°, E 35.290041°) and Samama community from Mangochi District on southern shoreline of Lake Malawi (S 14.418767°, E 35.220985°) (Kayuni et al. 2024b) (Figure 1).

This sub-study population comprised of all women aged 18 years and above, who were sexually active, non-pregnant, not currently menstruating, with a proven or suspected interspecies *Schistosoma*-hybrid or zoonotic infection upon molecular analysis of schistosome eggs in urine at the main HUGS Human Baseline survey in June 2022 (Supplementary Figure S1). The participants were recruited at baseline of the FGS sub-study in June 2023 and underwent a follow-up at 6 months and then at 12 months, with single-dose praziquantel treatment (40 mg/kg) offered after each inspection. As this study was principally a pilot study to detect zoonotic and hybrid schistosomes, a minimum sample size of 50 women with proven UGS was judged appropriate.

### Data collection

All participants were interviewed using a structured questionnaire covering demographical details, recent travel, water contact and anthelminthic treatment history after giving an informed written consent (Supplementary Figure S2).

### Urine and stool microscopy

A 120-mL clear container was used to collect a urine sample during the study visit, which was then filtered and examined microscopically to detect *Schistosoma* eggs in 10 mL of the well-mixed sample (Cheesbrough, 2005). Each sample was also tested using reagent dipstick and point-of-care circulating cathodic antigen. Additionally, where provided, stool samples were collected and analysed using Kato–Katz technique to detect *Schistosoma* eggs.

Thereafter, the participants underwent further evaluations including colposcopy, cervicovaginal lavage (CVL) microscopy and molecular analysis, together with molecular analysis of cervicovaginal swab.

### FGS diagnosis by visual inspection (colposcopy)

The participant underwent visual inspection of cervicovaginal areas by colposcopy. The gynaecologist and midwives captured images of the cervix, fornices and vagina using a hand-held colposcope (EVA MobileODT) (Kayuni et al. 2024a). Images were evaluated on site by the gynaecologist and classified as *visual-FGS* if homogeneous sandy patches, grainy sandy patches (GSP), rubbery papules (RP) or ABV were present, and negative if none were observed (WHO, 2015).

### FGS diagnosis by parasitological methods

The midwives inserted a lubricated speculum and collected a cervicovaginal sample using a cotton tipped swab. Swabs were placed in individual screw cap microtubes and preserved in 1 mL of 70% ethanol. Following this, a CVL was obtained by flushing the vaginal walls and cervix with 10 mL of normal saline for a minute and the lavage collected from the posterior fornix with a pipette into 15-mL conical Falcon tubes. These samples were examined as wet mount under a microscope before centrifugation and microscopy of the sediments. The results were classified and recorded as *parasitological-FGS*. The remaining sediments were preserved in a microtube with 1 mL of 70% ethanol for molecular analysis at Liverpool School of Tropical Medicine (LSTM) in the United Kingdom (Cunningham et al. 2024).

### FGS diagnosis by molecular methods

As previously described (Cunningham et al. 2024; Kayuni et al. 2024a), molecular analysis of the CVL sediments and genital swabs included High-Resolution Melt and TaqMan real-time PCR for *Schistosoma* spp. Any Ct-value observed was classified as *molecular-FGS* positive. HPV markers were also screened for using the QIAscreen HPV PCR Test kit (Qiagen, Manchester UK), capable of screening for 2 high-risk genotypes, 16 and 18 alongside the others.

### FGS diagnosed by tissue examination

Women who were observed to have visible abnormal lesions on colposcopy were further requested for a punch biopsy of the lesion. Part of this tissue was examined directly by microscopy by squashing between 2 glass slides and the remaining tissue collected from the biopsy was sent for histopathological examination in a pathology laboratory in Blantyre where any other conditions/diseases, in addition to schistosome eggs, were noted. Presence of *Schistosoma* DNA in the tissue biopsy also examined to confirm the diagnosis of FGS and identity of the schistosome species.

### Data analyses

The data collected were entered into Kobo Toolbox and then transferred to *Microsoft Excel 2021* package for cleaning and summary statistics. *SPSS version 28.0* package was then utilized for further statistical analyses. Participant demographics were summarized by median and range for continuous variables and by frequency and percentage for categorical variables. The primary outcome measured includes the infection status for FGS and other genital diseases including HPV, and whether the presence of FGS affects acquisition of other diseases. This was analysed using field microscopy, laboratory real-time PCR, and comparing the prevalence of FGS and HPV across both years. Secondary outcomes include age prevalence rates and the geographical distribution of schistosomiasis and HPV. This was to determine the population at most risk for each disease and whether there is an overlap of the distribution of positive infections.

Continuous variables such as age and intensity of infection in urine/CVL/biopsy have been transformed into categorical variables for further statistical analysis. Overall, results are presented in graphs and summary tables. Survey results are reported as a total prevalence and further categorized into causative pathogens, geography, infection intensity and age. Prevalence data was computed with 95% confidence intervals (CI). Crude odds ratios are reported with the 95% CI and *P*-value. Geographical mapping was also done to assess the distribution of infections divided into both survey sites (Nsanje and Mangochi).

The Clopper–Pearson’s exact test was used to acquire the CI. Statistical significance was set at a *P*-value of <0.05. Chi squared test was carried out to compare infection rates within each time point. The Wilcoxon’s rank sum test was used to compare ordinal or continuous data such as the intensity of infection within each time point. The paired Cochran *Q* was used to determine significance of an increase or decrease, i.e. prevalences at the different time points.

### Ethical considerations

Ethical approval for the study was granted by the College of Medicine Research Ethics Committee (COMREC), Kamuzu University of Health Sciences (KUHeS), Malawi, (Approval number: P.08/21/3381) and the LSTM Research Ethics Committee (LSTM REC) in the United Kingdom (registration number: 22-028). Informed consent to participate in the study was obtained from all participants. Privacy and confidentiality were maintained throughout the study.

## Results

A total of 86 participants were recruited into the FGS sub-study at baseline in June 2023 (28 in Nsanje District, 58 in Mangochi District), 64 (16 in Nsanje, 48 in Mangochi) and 81 (25 in Nsanje, 56 in Mangochi) were present at 6- and 12-months’ follow-up time points respectively. The median age at baseline was 27.0 years (range: 18–49), 28.0 years in Nsanje (range: 19–41) and 25.0 in Mangochi (range: 18–49) (Table 1). Forty-two participants (48.8%) were aged 25 years and below, 56 were married (65.1%), 59 had attained primary education (68.6%) and 41 were farmers (47.7%).

**Table 1.** Demographical characteristics of the study participants at Baseline

Variable	Category	Total	Nsanje	Mangochi
Age (years)	18–25	40	10	30
	26–32	21	9	12
	33–39	18	8	10
	40+	7	1	6
Marital status	Single	19	4	15
	Married	56	21	35
	Divorced/Separated	9	3	6
	Widowed	2	0	2
	Other	0	0	0
Tribe	Chewa	40	0	40
	Yao	9	0	9
	Sena	28	28	0
	Mang’anja	4	0	4
	Other	5	0	5
Religion	Christian	82	28	54
	Muslim	4	0	4
Education	None	16	9	7
	Primary	59	16	43
	Secondary	8	1	7
	Tertiary	3	2	1
Occupation	Student	2	1	1
	Farmer	41	26	15
	Business	17	1	16
	Unemployed	22	0	22
	Other	4	0	4

At 6 months’ follow-up, the median age was 28.0 years (range: 18–49), 30.0 years in Nsanje (range: 18–49) and 24.5 in Mangochi (range: 18–49) and at 12 months, 25.0 years (range: 18–50), 27.0 years in Nsanje (range: 18–47) and 25.0 in Mangochi (range: 18–50) (Supplementary Table S1).

**Figure 2.**
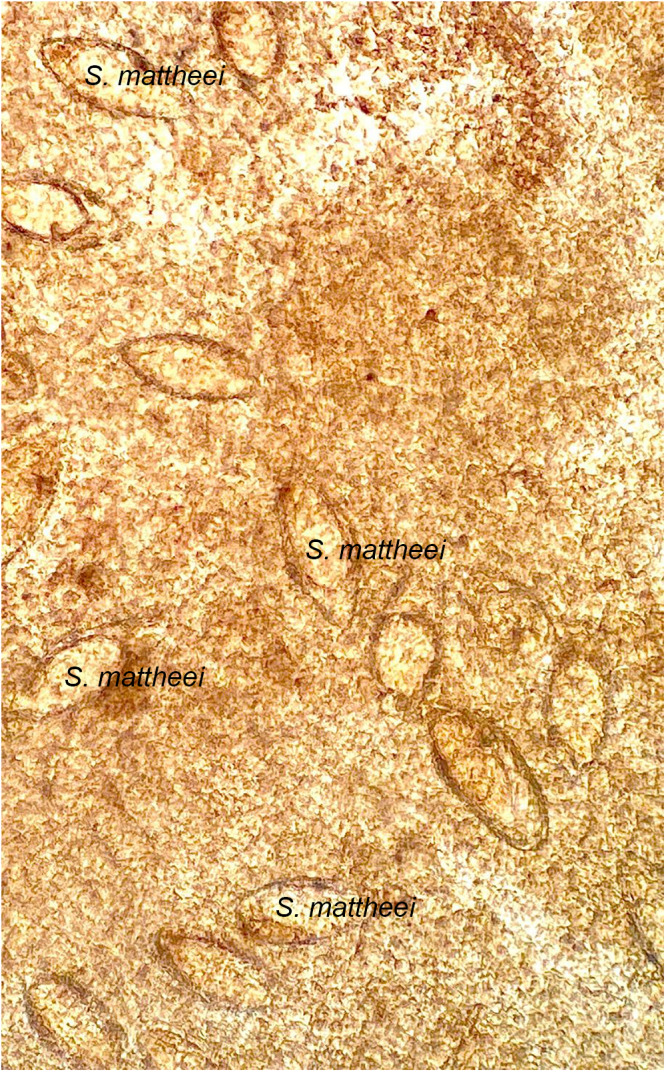
Numerous schistosome eggs within a CVL with some showing atypical morphology resembling *S. mattheei.* (Image courtesy of Professor JR Stothard).

**Figure 3.**
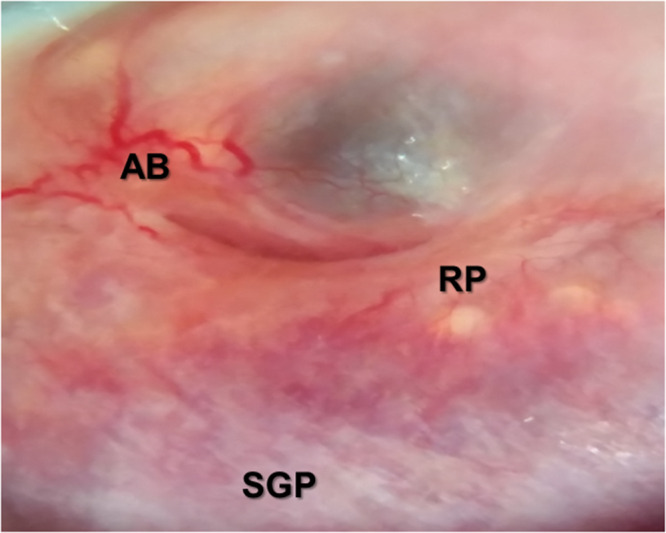
Image from the portable MobileODT EVA COLPO showing different FGS lesions, namely sandy grainy patches (*SGP*), abnormal vessels and bleeding (*AB*), and rubbery papules (*RP*) detected on colposcopy. Magnification, ×20. (Image courtesy of Dr. Dingase Kumwenda). (Kayuni *et al.* 2024a).

At baseline, 34 participants (42.5%) had *Schistosoma* eggs in their urine, 14 (56.0%) in Nsanje and 20 (36.4%) in Mangochi (Table 2). Twenty-five participants (29.1%) had eggs detected in CVL upon microscopy of the wet mount preparation, of whom 9 (31.0%) were from Nsanje with 1 having *S. mattheei* eggs and 16 (10.3%) in Mangochi (Figure 2). Sixty-two women (72.1%) were FGS positive by colposcopy at baseline, 22 (78.6%) in Nsanje and 40 (69.0%) in Mangochi. The commonest presentation was homogeneous yellow, sandy patches, followed by GSP (Figure 3).

**Table 2.** Baseline prevalence of FGS and HPV infection among the study participants using different diagnostic tests

Variable	Category	Total	Nsanje	Mangochi
Visual – FGS	Homogenous yellow sandy patches	62	22	40
	Grainy sandy patches	15	4	11
	Rubbery papules	5	1	4
	Abnormal blood vessels	11	5	6
	Overall	62 (72.1%)	22 (78.6%)	40 (69.0%)
Parasitological – FGS	CVL	25 (29.1%)	9 (31.0%)	16 (10.3%)
Molecular – FGS	CVL	44	18	26
	Swab	34	15	19
	Overall	54 (64.3%)	21 (75.0%)	33 (69.0%)
HPV	CVL	12	2	10
	Swab	26	9	17
	Overall	26 (31.0%)	9 (32.1%)	17 (30.4%)
Parasitological – UGS	Urine	34 (42.5%)	14 (56.0%)	20 (36.4%)
Visual FGS vs Molecular FGS vs HPV	V+ M+	43 (51.2%)	16	27
	V+ M–	16	5	11
	V– M+	10	4	6
	V– M–	15	3	12
	All (V+ M+ H+)	14 (8.3%)	7 (25.0%)	7 (12.5%)

FGS, female genital schistosomiasis; HPV, Human Papilloma Virus; V+, visual – FGS (FGS positive by colposcopy); M+, molecular – FGS (FGS positive by PCR); H+,  HPV positive by PCR; UGS, urogenital schistosomiasis.

A total of 54 women (64.3%) were FGS positive by molecular PCR analysis at baseline, 21 (75.0%) in Nsanje and 33 (69.0%) in Mangochi. Forty-three women (51.2%) were FGS positive by both colposcopy and real-time PCR. Human papillomavirus (HPV) was detected in 26 women (31.0%), showing high-risk genotypes 16 and 18, associated with cervical cancer. Interestingly, 14 women (8.3%) were FGS positive by colposcopy and real-time PCR with detectable HPV.

Twenty-six participants (30.2%) reported symptoms associated with FGS at baseline, namely abnormal vaginal discharge, coital pain, post-coital vaginal bleeding and genital sores, 9 in Nsanje (32.1%) and 17 in Mangochi (29.3%). Twenty of the 42 participants (47.1%) who were FGS positive by colposcopy had those FGS-related symptoms, 7 in Nsanje (31.8%) and 13 in Mangochi (25.0%).

Sixteen cervical tissue biopsies conducted at baseline, each from 3 participants in Nsanje and 13 in Mangochi, showed *Schistosoma* eggs on histopathological examination, confirming their diagnosis of FGS. Of these participants, only 11 (2 in Nsanje and 9 in Mangochi) were concurrently positive on real-time PCR.

At 6 months’ follow-up, 8 participants (13.6%; 2 (16.7%) in Nsanje and 6 (13.6%) in Mangochi) had *Schistosoma* eggs in CVL microscopy on wet mount preparation. Of these, 1 had *S. mattheei* eggs and *Trichomonas vaginalis* in the CVL as well as *S. haematobium* eggs on crushed biopsy. Thirty-four women (65.4%) were FGS positive by coloscopy, 10 (62.5%) in Nsanje and 24 (50.0%) in Mangochi, showing a reduction in the FGS prevalence after PZQ treatment (Table 3). Similarly, 23 participants (39.0%; 3 (20.0%) in Nsanje and 20 (45.5%) in Mangochi) were FGS positive by real-time PCR, while HPV was detected in only 11 women (18.6%; all 11 (25.0%) from Mangochi).

**Table 3.** Results of the different diagnostic tests for FGS and HPV conducted on the study participants at the 3 time points

Variable		Baseline		6 months		12 months	
		Nsanje	Mangochi	Nsanje	Mangochi	Nsanje	Mangochi
Visual – FGS	*N* (*n*)	22 (28)	40 (58)	10 (16)	24 (48)	11 (25)	52 (56)
	%	78.6	69.0	62.5	50.0	44.0	92.9
Molecular – FGS	*N* (*n*)	21 (28)	33 (56)	3 (15)	20 (44)	2 (18)	5 (51)
	%	75.0	69.0	20.0	45.5	11.1	9.8
HPV	*N* (*n*)	9 (28)	17 (56)	0 (15)	11 (44)	1 (18)	13 (51)
	%	32.1	30.4	0.0	25.0	5.6	25.5

FGS, female genital schistosomiasis; HPV, Human Papilloma Virus; Visual – FGS, FGS positive by colposcopy; Molecular – FGS, FGS positive by real-time PCR.

At 12 months’ follow-up, 11 women (44.0%) in Nsanje were FGS positive by coloscopy, a further reduction in the FGS prevalence compared to a substantial rise in the number of women in Mangochi to 52 women (92.9%), giving an overall prevalence of 77.8% at this time point. Three participants (3.7%; only 5.8% in Mangochi) had *Schistosoma* eggs in CVL microscopy. However, 7 women (10.1%; 2 (11.1%) in Nsanje and 5 (9.8%) in Mangochi) were FGS positive by real-time PCR. HPV was detected in 14 women (20.3%; 1 (5.6%) in Nsanje and 13 (25.5%) in Mangochi).

**Figure 4.**
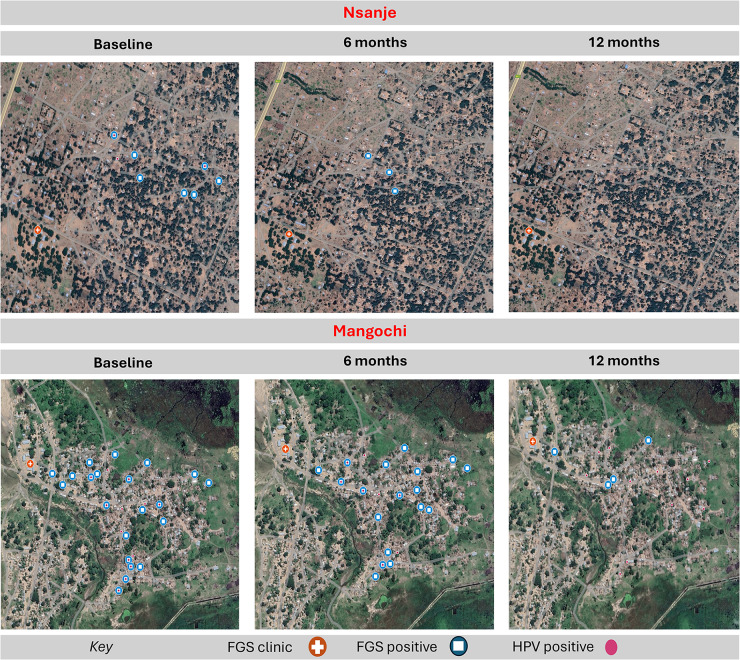
Geospatial plot of positive FGS infections and their urine egg-patent occurrence.

Fifty-two participants (10 in Nsanje and 42 in Mangochi) were present for examination in the study at all the 3 time points. Among these participants, 47 (90.4%; 9 (90.0%) in Nsanje and 38 (90.5%) in Mangochi) had *Schistosoma* eggs in their urine at baseline, which reduced to 16 (30.8%; 3 (30.0%) in Nsanje and 13 (31.0%) in Mangochi) at 6 months’ follow-up and further to 4 participants (7.7%; 2 (20.0%) in Nsanje and 2 (4.8%) in Mangochi) at 12 months’ follow-up (Table 4 and Figure 4).

**Table 4.** Prevalence of urine egg-patent UGS among 52 FGS participants present at all the 3 time points across the study sites

Study site	Positive at baseline *N* (%)	Positive at 6 months *N* (%)	Positive at 12 months *N* (%)
Nsanje	9 (90.0)	3 (30.0)	2 (20.0)
Mangochi	38 (90.5)	13 (31.0)	2 (4.8)
All	47 (90.4)	16 (30.8)	4 (7.7)

Similar to the results above, 27 participants (51.9%; 6 (60.0%) in Nsanje and 21 (50.0%) in Mangochi) were FGS positive by coloscopy at baseline (Table 5). Despite the overall FGS prevalence by colposcopy lesions increasing by 32.7% over the year follow-up to 84.6%, the prevalence among Nsanje participants declined from 60.0% to 50.0%.

**Table 5.** Overall prevalence of FGS from colposcopy examinations among the 52 study participants at the 3 time points across the study sites

Study site	Positive at baseline *N* (%) [CI] *P* value	Positive at 6 months *N* (%) [CI] *P* value	Positive at 12 months *N* (%) [CI] *P* value
Nsanje	6 (60)	6 (60)	5 (50)
	[26.2−87.8]	[26.2−87.8]	[18.7−81.3]
	0.527	0.527	1
Mangochi	21 (50)	22 (52.4)	39 (92.9)
	[34.2–65.8]	[36.4−68]	[80.5−98.5]
	1	0.758	<0.001
All	27 (51.9)	28 (53.8)	44 (84.6)
	[37.6−66]	[39.5−67.8]	[71.9−93.1]
	0.782	0.579	<0.001

The most common lesion observed on colposcopy across all time points was homogenous yellow sandy patches (HYP) which affected 50% or more, followed by GSP (Table 6). The HYPs increased by 34.6% at end of the survey, likewise to other pathologies GSP, abnormally dilated blood vessels and RP (Figure 3).

**Table 6.** Overall colposcopy findings among the 52 study participants at the 3 time points across the study sites

Pathological findings	Baseline *N* (%) [CI] *P* value	6 months *N* (%) [CI] *P* value	12 months *N* (%) [CI] *P* value
Homogenous yellow sandy patches	26 (50)	26 (50)	44 (84.6)
	[35.8–64.2]	[35.8–64.2]	[71.9−93.1]
	1	1	<0.001
Grainy sandy patches	7 (13.5)	12 (23.1)	16 (30.8)
	[5.6–25.8]	[12.5 6.8]	[18.7 5.1]
	<0.001	<0.001	0.006
Abnormal blood vessels	8 (15.4)	5 (9.6)	8 (15.4)
	[6.9−28.1]	[3.2−21]	[6.9−28.1]
	<0.001	<0.001	<0.001
Rubbery papules	3 (5.8)	6 (11.5)	7 (13.5)
	[1.2−15.9]	[4.4−23.4]	[5.6−25.8]
	<0.001	<0.001	<0.001

**Figure 5.**
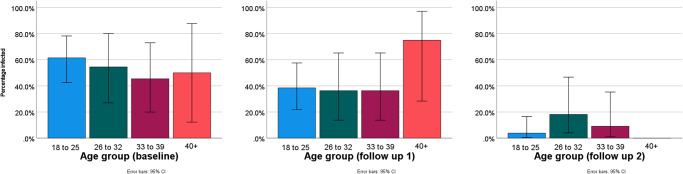
Prevalence of FGS according to age across both survey sites from colposcopy results.

According to age, 14 of the 27 participants (51.9%) with FGS colposcopy lesions were in the youngest age group of 18–25 years at baseline which was the most affected during the study (Table 7 and Figure 5). Notably, almost all of those within the oldest age group of 40 years and above had lesions across all time points.

**Table 7.** Prevalence of FGS by colposcopy among the 52 study participants according to age across the study sites

Age group	Total sampled *N*	Positive at Baseline *N* (%) [CI] *P* value	Total sampled *N*	Positive at 6 months *N* (%) [CI] *P* value	Total sampled *N*	Positive at 12 months *N* (%) [CI] *P* value
18−25	26	14 (53.8)	26	10 (38.5)	26	22 (84.6)
		[33.4−73.4]		[20.2−59.4]		[65.1−95.6]
		0.695		0.239		<0.001
26−32	11	6 (54.5)	11	6 (54.5)	11	9 (81.8)
		[23.4−83.3]		[23.4−83.3]		[48.2−97.7]
		0.035		0.763		0.763
33–39	11	4 (36.4)	11	8 (72.7)	11	9 (81.8)
		[10.9−69.2]		[39−94]		[48.2−97.7]
		0.366		0.132		0.035
40+	4	3 (75)	4	4 (100)	4	4 (100)
		[19.4−99.4]		[39.8−100]		[39.8−100]
		0.317		0.125		0.125

Any observed colposcopy findings except for HYP results at baseline and 6 months’ follow-up were significant, *Χ*² (*P* < 0.05). The observed proportion of participants infected was not significant for most results *Χ*^2^ (*P* > 0.05) except for those infected in Mangochi and overall infection rates during the 12 months’ follow-up. In terms of age, only the first 3 age groups during the 12 months’ follow-up had a statistically significant difference *Χ*^2^ (*P* > 0.05). The increase in prevalence of FGS according to colposcopy lesions across the 3 time points was statistically significant *Χ*^2^ (2) = 16.5 (*P* < 0.001).

Seven participants (13.5%; 3 (30.0%) were from Nsanje and 4 (9.5%) were from Mangochi; *P* < 0.001) had *Schistosoma* eggs detected on parasitological examination of the CVL and crushed biopsies at baseline which increased by 11.5% to 13 participants (25.0%; 2 (20.0%) were from Nsanje and 11 (26.2%) were from Mangochi; *P* < 0.001) at 12 months’ follow-up (Table 8). Prevalence of FGS by CVL microscopy was highest in the youngest age group (18–25) at 19.2%, 15.4% and 23.1% during baseline, 6- and 12-months’ follow-up (Table 9). The oldest age group (40+ years) had no infections throughout the study period. Only the youngest age group (18–25) had a statistically significant difference in the FGS prevalence by CVL tests, *Χ*^2^ (*P* > 0.05) across all time points.

**Table 8.** Overall prevalence of FGS from combining cervicovaginal lavages and biopsy results

Study site	Total sampled *N*	Positive at Baseline *N* (%) [CI] *P* value	Total sampled *N*	Positive at 6 months *N* (%) [CI] *P* value	Total sampled *N*	Positive at 12 months *N* (%) [CI] *P* value
Nsanje	**10**	3 (30.0)	**10**	2 (20.0)	**10**	2 (20)
		[6.7−65.2]		[2.5−55.6]		[2.5−55.6]
		0.206		0.058		0.206
Mangochi	**42**	4 (9.5)	**42**	6 (14.3)	**42**	11 (26.2)
		[2.7−22.6]		[5.4−28.5]		[13.9−42]
		0.002		<0.001		<0.001
Total	**52**	**7 (13.5)**	**52**	**8 (15.4)**	**52**	**13 (25)**
		**[5.6–25.8]**		**[6.9−28.1]**		**[14−38.9]**
		**<0.001**		**<0.001**		**<0.001**

**Table 9.** Prevalence of FGS according to age across both survey sites from combined CVL and biopsy results

Age group	Total sampled *N*	Positive at baseline *N* (%) [CI] *P* value	Total sampled *N*	Positive at 6 months *N* (%) [CI] *P* value	Total sampled *N*	Positive at 12 months *N* (%) [CI] *P* value
18−25	26	5 (19.2)	26	4 (15.4)	26	6 (23.1)
		[6.6−39.4]		[4.4−34.9]		[9−43.6]
		0.002		<0.001		0.006
26−32	11	1 (9.1)	11	4 (36.4)	11	2 (18.2)
		[0.2−41.3]		[10.9−69.2]		[2.3−51.8]
		0.007		0.366		0.035
33−39	11	1 (9.1)	11	0 (0)	11	5 (45.5)
		[0.2−41.3]		[0−28.5]		[16.7−76.6]
		0.007		*Not calculated		0.763
40+	4	0 (0)	4	0 (0)	4	0 (0)
		0−60.2		0−60.2		0−60.2
		*Not calculated		*Not calculated		*Not calculated

On molecular analysis of the CVL and swab, FGS infection rates decreased by 48.1% over study period with majority of Nsanje and Mangochi participants having FGS at baseline and observed a 70% and 42.9% reduction respectively by the end of the survey (Table 10). Most of those infected are from the youngest age group (18–25) with 61.5%, 38.5% and 3.8% infected at all the 3 time points. Any observed overall prevalence was only significant, *Χ*^2^ (*P* < 0.05) at 12 months’ follow-up, including age groups (18–25, 26–32 and 33–39). Cochran’s *Q* test indicates that the decrease in the overall prevalence of FGS according to both CVL and swab results across the 3 time points is statistically significant *Χ*^2^ (2) = 29.6 (*P* < 0.001).

**Table 10.** Prevalence of FGS according to age across both survey sites from combined CVL and swab PCR results

Age group	Total sampled *N*	Positive at Baseline *N* (%) [CI] *P* value	Total sampled *N*	Positive at 6 months *N* (%) [CI] *P* value	Total sampled *N*	Positive at 12 months *N* (%) [CI] *P* value
18−25	26	16 (61.5)	26	10 (38.5)	26	1 (3.8)
		[40.6−79.8]		[20.2−59.4]		[0.1−19.6]
		<0.001		0.239		0.239
26−32	11	6 (54.5)	11	4 (36.4)	11	2 (18.2)
		[23.4−83.3]		[10.9−69.2]		[2.3−51.8]
		0.763		0.366		0.035
33–39	11	5 (45.5)	11	4 (36.4)	11	1 (9.1)
		[16.7−76.6]		[10.9−69.2]		[0.2−41.3]
		0.763		0.366		0.007
40+	4	2 (50)	4	3 (75)	4	0 (0) [0−60.2]
		[6.8−93.2]		[19.4−99.4]		
		1		0.317		*Not calculated

**Figure 6.**
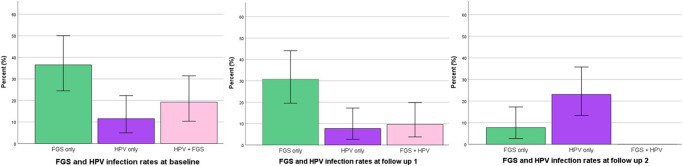
Prevalence of FGS-HPV coinfection from combined real-time PCR of CVL and swab.

With regards to HPV, the PCR results showed that a prevalence was 30.8% at baseline which decreased by 7.7% over 1 year, with an observed higher decline in 6 months compared to 12 months’ follow-up possibly due to the transient nature of the disease (Tables 11, 12 and Figure 6). Most of those infected with HPV are from the youngest age group (18–25), 38.5% at baseline, 26.9% at 6 months’ and 15.4% at 12 months’ follow-up. Any observed overall prevalence was significant, *Χ*^2^ (*P* < 0.05) at all study time points.

Replace this Figure 6 with the one attached, **Figure 6_new**

**Table 11.** Prevalence of HPV 16/18 according to age across both study sites from combined CVL and swab PCR results

Age group	Total sampled *N*	Positive at baseline *N* (%) [CI] *P* value	Total sampled *N*	Positive at 6 months *N* (%) [CI] *P* value	Total sampled *N*	Positive at 12 months *N* (%) [CI] *P* value
18−25	26	10 (38.5)	26	7 (26.9)	26	4 (15.4)
		[20.2−59.4]		[11.6−47.8]		[4.4−34.9]
		0.239		0.019		<0.001
26−32	11	3 (27.2)	11	1 (9.1)	11	5 (45.5)
		[6−61]		[0.2−41.3]		[16.7−76.6]
		0.132		0.007		0.763
33–39	11	3 (27.3)	11	1 (9.1)	11	2 (18.2)
		[6−61]		[0.2−41.3]		[2.3−51.8]
		0.132		0.007		0.035
40+	4	0 (0)	4	0 (0)	4	1 (25)
		[0−60.2]		[0−60.2]		[0.6−80.6]
		*Not calculated		*Not calculated		0.317

**Table 12.** Prevalence of FGS-HPV coinfection from combined PCR of CVL and swabs

Variable	Baseline		6-months		12-months	
	HPV positive (*N*)	HPV Negative (*N*)	HPV Positive (*N*)	HPV Negative (*N*)	HPV Positive (*N*)	HPV Negative (*N*)
FGS positive (*N*)	10	19	5	16	0	4
FGS negative (*N*)	6	17	4	27	12	36
All	16	26	9	43	12	40

## Discussion

As previously described, FGS remains underdiagnosed in schistosomiasis-endemic areas across SSA (Swai et al. 2006; Bustinduy et al. 2022; Lamberti et al. 2024b) and particularly in Southern Malawi where this longitudinal cohort study was conducted. Our study has also confirmed the unique detection of a zoonotic schistosome infection, *S. mattheei*, alongside the more dominant human schistosome *S. haematobium* in the CVL, cervical tissue biopsies collected and on real-time PCR (Kayuni et al. 2024a; Stothard et al. 2025). Infection with this schistosome was reported in an intriguing case in Nsanje which illustrated the diagnostic discordance between detection methods for active UGS and chronic FGS.

*Schistosoma mattheei* is a common parasite of livestock and is known to infect people (Díaz et al. 2023). Here in Malawi, local infections of *S. mattheei* in cattle can be common with many people within our study cohort observed to share water contact points with these animals (Juhász et al. 2024a, b). Such communal water bodies can harbour competent intermediate snail hosts for both human and zoonotic species of schistosomes, thereby transmitting the worms between humans and nearby livestock (Stothard et al. 2020). The presence of zoonotic infection within FGS poses a new challenge to the current disease control strategies of the Malawi Ministry of Health, National Schistosomiasis Control Programme which are focussed on annual mass drug administration with PZQ to people living only in areas with high and moderate burdens. Hence, there is now a need to better understand the pathology of zoonotic and hybrid schistosomes particularly in the context of One Health future efforts to eliminate schistosomiasis as a public health concern (WHO, 2020, 2022).

Our study among a relatively young population of women aged 18–49 years (median age: 28 years) detected a baseline prevalence of FGS among the 86 study participants in mid-2023 to range from 29.1% (*parasitological–FGS*, on CVL microscopy), 64.3% (*molecular-FGS*, by PCR) to 72.1% (*visual-FGS*, by colposcopy). A recent study completed in the same region in Malawi revealed an extensive burden of FGS detected via clinical colposcopy alone, as was used in this case study, noting a prevalence of 21.5% compared with 6.8% upon egg-patent urine microscopy as a proxy for FGS diagnosis (Lamberti et al. 2024a). Our study reported that 42.5% participants had *Schistosoma* egg-patent urine at baseline which is similarly lower than diagnosis by either colposcopy or PCR.

Urine microscopy, the foundational method to detect UGS, has been commonly used as a diagnostic proxy for FGS in most endemic areas with limited capacity (Christinet et al. 2016; Makia et al. 2023). However, it is known that *Schistosoma* eggs can present in cervicovaginal fluid (Swart and van der Merwe, 1987), with their absence in urine, as seen in our study (Poggensee et al. 1998). Additionally, our earlier case report from this study cohort described application of several diagnostic techniques to detect or incriminate FGS where most of the definite standard tests (urine filtration, CVL microscopy and real-time PCR) each produced a negative result, relying on other techniques like colposcopy (Kayuni et al. 2024a).

Of note, our study cohort for FGS had UGS at baseline which declines during follow-up after PZQ treatment, many FGS-positive participants had low intensity or absent UGS at 6 and 12 months which further validates that FGS can exist without UGS and can be easily missed if only urine samples are analysed. Furthermore, although molecular techniques have advanced diagnostic capability and high detection rate (Cunningham et al. 2018), real-time PCR did not detect any schistosome genetic material in the CVL despite the colposcopy examination revealing classical lesions of FGS, namely sandy grainy patches, ABV and RP (WHO, 2015). These may be old pathologies which are not harbouring active, live schistosome eggs, releasing genetic material. Certainly, the visible lesions which had biopsies performed from, may underestimate subclinical lesions arising from FGS, which highlights the need for a range of techniques including clinical history of the symptoms and physical examination, parasitological and molecular tests among others.

These lesions have also been observed to increase the susceptibility to HPV and HIV as well as cervical cancer (Hotez et al. 2019; Patel et al. 2021). Hence, the need for improved availability and accessibility of PZQ treatment to all diagnosed and suspected cases in endemic areas as such FGS – HPV coinfection can result in an increase the morbidity of the infections and further reduces quality of life. This correlates with prior reports of the ability of the PZQ drug to improve patients’ well-being beyond the direct cure of the disease (Zwang and Olliaro, 2014; Bustinduy et al. 2022; Wiegand et al. 2022). In a related manner, we demonstrate the reduction of the prevalence of FGS using some techniques, highlighting the importance of PZQ.

From our findings, we advocate for the use of multiple diagnostic techniques in addition to a thorough patient history, water-contact behaviours and pelvic examination to improve FGS diagnosis. Also, health education (inclusive of One Health components), raised community awareness, improved availability and accessibility of PZQ and synergistic strategies and integration of genital schistosomiasis services in existing services in Sexual Reproductive Health and Rights, HIV/AIDS and other health programmes in the Ministry of Health would greatly assist in understanding, management and holistic control interventions of FGS, HPV and other infection in the country and region.

In conclusion, here in southern Malawi, our study describes FGS as a prevalent chronic manifestation of UGS, mainly caused by *S. haematobium* but also includes *S. mattheei*, a zoonotic species. Coinfection with other genital infections like high-risk HPV genotypes was confirmed highlighting the complexity of dual FGS – HPV diagnosis, treatment and management, where portable colposcopy and real-time PCR technologies may not be available.

## Supporting information

Kumwenda et al. supplementary material 1 Kumwenda et al. supplementary material

Kumwenda et al.supplementary material 2 Kumwenda et al.supplementary material

Kumwenda et al.supplementary material 3 Kumwenda et al.supplementary material

## Data Availability

The datasets generated and analyzed for this study have been included in this manuscript.

## Supplementary material

The supplementary material for this article can be found at https://doi.org/10.1017/S0031182025100802
