# Supplementary material for: An investigation of female genital schistosomiasis and associated genital infections in Southern Malawi
Source: Parasitology. 2025 Sep 1;152(14):1508–19. doi: 10.1017/S0031182025100802 (PMC13124304; doi:10.1017/S0031182025100802)
Supplement: Kumwenda et al. supplementary material 1 — Kumwenda et al. supplementary material [file S0031182025100802sup001.docx]

**Supplementary Table**

**Table S1: Age distribution of the study participants at the 3 time points in comparison with results of different diagnostic tests**

| **Study area** | **Variable** | | **Baseline** | | **6-months** | | **12-months** | |
| --- | --- | --- | --- | --- | --- | --- | --- | --- |
|  |  |  | **Age** | **Ct-value** | **Age** | **Ct-value** | **Age** | **Ct-value** |
| *Nsanje* | *Visual – FGS* | *Median* | 27.0 | - | 28.5 | - | 36.0 | - |
|  |  | *Range* | 19.0 – 38.0 | - | 20.0 – 37.0 | - | 22.0 – 43.0 | - |
| *Mangochi* |  | *Median* | 25.0 | - | 31.0 | - | 25.0 | - |
|  |  | *Range* | 18.0 – 45.0 | - | 18.0 – 49.0 | - | 18.0 – 50.0 | - |
| *Nsanje* | *Molecular – FGS: All* | *Median* | 28.0 | - | 30.0 | - | 31.0 | - |
|  |  | *Range* | 19.0 – 41.0 | - | 20.0 – 37.0 | - | 19.0 – 43.0 | - |
| *Mangochi* |  | *Median* | 25.0 | - | 26.6 | - | 25.0 | - |
|  |  | *Range* | 18.0 – 45.0 | - | 18 – 42 | - | 19.0 – 35.0 | - |
| *Nsanje* | *Molecular – FGS: CVL* | *Median* | - | 26.8 | - | 26.8 | 19.0 | 33.5 |
|  |  | *Range* | - | 18.9 – 36.4 | - | 25.8 – 28.6 | - | - |
| *Mangochi* |  | *Median* | - | 32.1 | - | 29.7 | - | 33.3 |
|  |  | *Range* | - | 17.6 – 36.8 | - | 20.7 – 38.1 | - | 23.3 – 35.1 |
| *Nsanje* | *Molecular – FGS: Swab* | *Median* | - | 26.1 | - | 26.3 | 43.0 | 36.7 |
|  |  | *Range* | - | 21.1 – 36.0 | - | 24.9 – 31.5 | - | 0 |
| *Mangochi* |  | *Median* | - | 27.1 |  | 28.7 | - | 30.9 |
|  |  | *Range* | - | 22.3 – 37.1 |  | 18.4 – 36.9 | - | 30.5 – 35.4 |
| *Nsanje* | *HPV: All* | *Median* | 26.0 | - | - | 0.0 | 29.0 | - |
|  |  | *Range* | 19.0 – 34.0 | - | - | 0.0 | - | - |
| *Mangochi* |  | *Median* | 25.0 | - | 23.0 | - | 30.0 | - |
|  |  | *Range* | 20.0 – 38.0 | - | 18.0 – 38.0 | - | 19.0 – 41.0 | - |
| *Nsanje* | *HPV – CVL* | *Median* | - | 23.0 | - | - | - | 35.3 |
|  |  | *Range* | - | 21.1 – 24.9 | - | - | - | - |
| *Mangochi* |  | *Median* | - | 27.8 | - | 27.3 | - | 32.4 |
|  |  | *Range* | - | 14.7 – 36.6 | - | 24.5 – 32.0 | - | 19.7 – 35.4 |
| *Nsanje* | *HPV – Swab* | *Median* | - | 28.3 | - | - | - | - |
|  |  | *Range* | - | 20.2 – 36.4 | - | - | - | - |
| *Mangochi* |  | *Median* | - | 25.3 | - | 30.6 | - | 24.9 |
|  |  | *Range* | - | 21.2 – 34.0 | - | 22.7 – 32.4 | - | 18.8 – 34.3 |
