# Supplementary figures and images for: An investigation of female genital schistosomiasis and associated genital infections in Southern Malawi
Source: Parasitology. 2025 Sep 1;152(14):1508–19. doi: 10.1017/S0031182025100802 (PMC13124304; doi:10.1017/S0031182025100802)

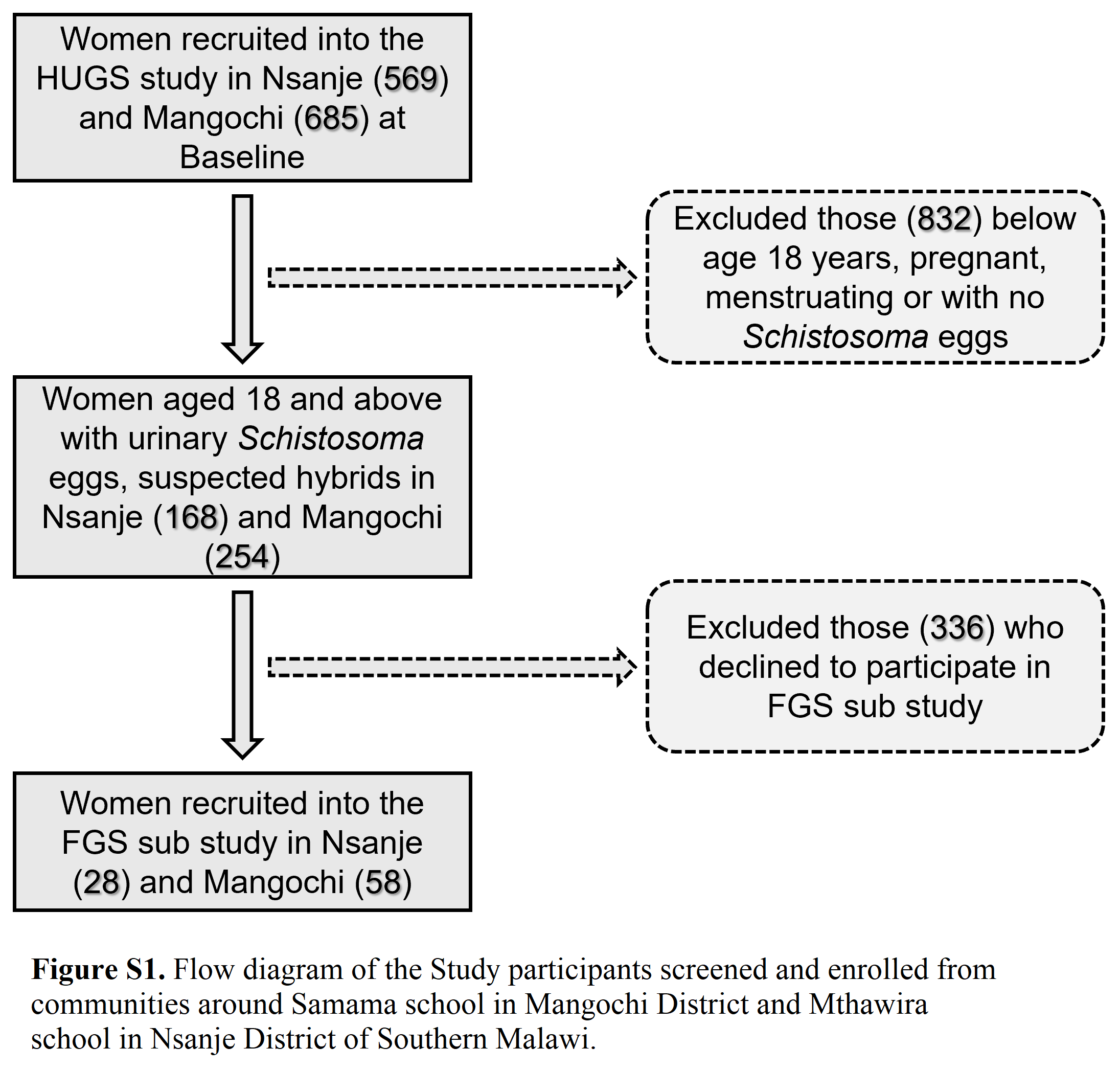

Supplement: Kumwenda et al.supplementary material 2 — Kumwenda et al.supplementary material [file S0031182025100802sup002.tiff]

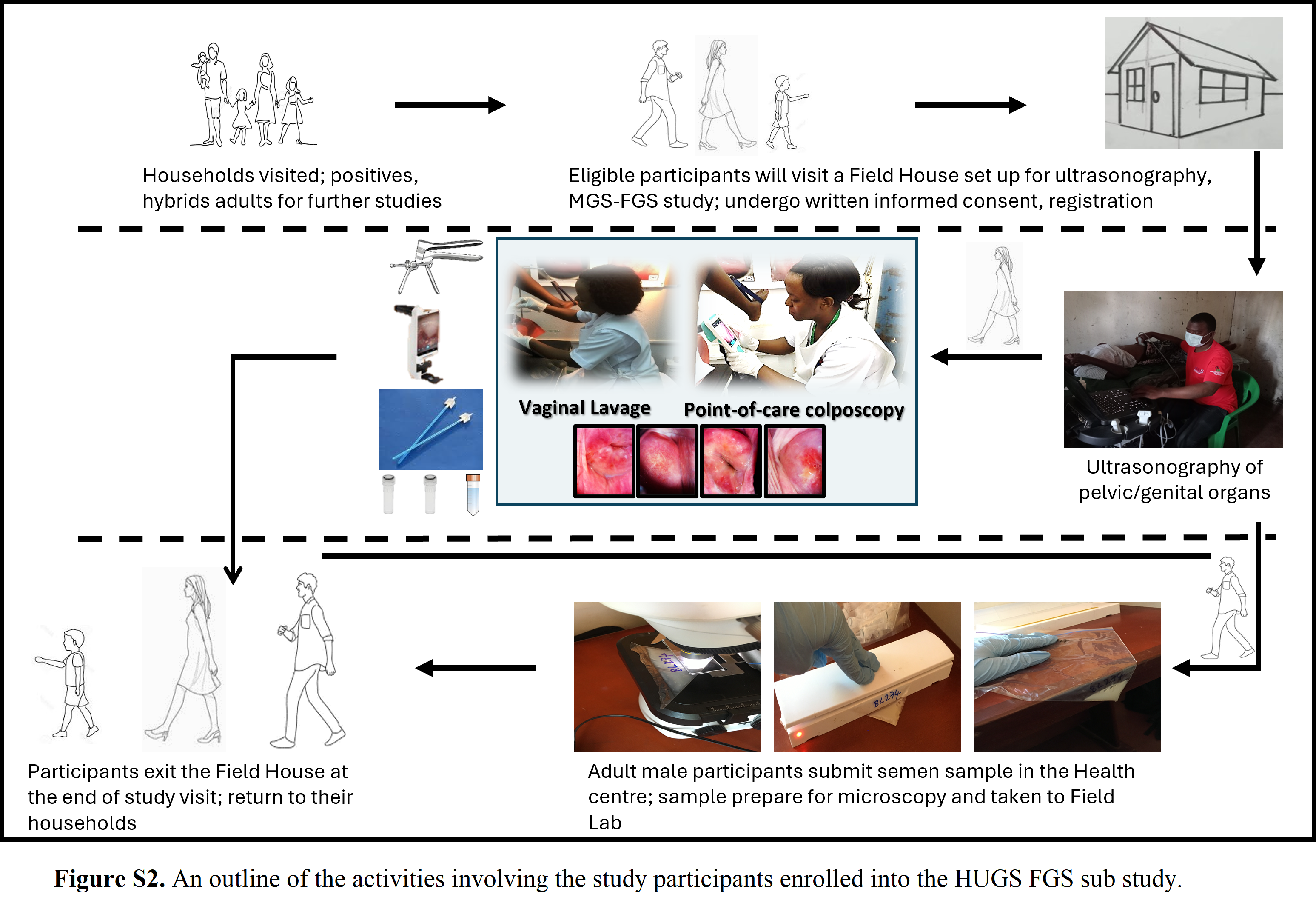

Supplement: Kumwenda et al.supplementary material 3 — Kumwenda et al.supplementary material [file S0031182025100802sup003.tiff]
